# Supplementary material for: Nkx2.9 Contributes to Mid-Hindbrain Patterning by Regulation of mdDA Neuronal Cell-Fate and Repression of a Hindbrain-Specific Cell-Fate
Source: Int J Mol Sci. 2021 Nov 23;22(23):12663. doi: 10.3390/ijms222312663 (PMC8658040; doi:10.3390/ijms222312663)
Supplement: Supplementary file 1 [file ijms-22-12663-s001.zip › SupplTableS1.pdf]

Gene name	Log2(FC)	Wald-stat p-value
Pcsk1n	0,4146166526	0,000048003
Gm7536	0,4054588154	0,0005670297
**En1**	**0,3996925462**	**0,0005576358**
Rnu1a1	0,3633070995	0,0028868715
Mt3	0,3550846834	0,0032496994
**Pax7**	**0,3500817405**	**0,0034758224**
Ndufa11	0,3481034644	0,0043853233
Gm15427	0,3383818254	0,0050271379
Ttc9b	0,3335230703	0,005896077
Snora78	0,3263346364	0,0073576189
**En2**	**0,3180848674**	**0,0031561926**
Evx1os	0,3171962712	0,0002457122
Vstm2l	0,3136170096	0,0044174195
Map3k10	0,2987768445	0,0038369143
Nomo1	0,296619302	0,0071867956
Evx2	0,2857290737	0,0082611348
Gm22748	0,2850035298	0,0041804413
Clta	0,2739191983	0,0099375215
**Tph2**	**0,2731224029**	**0,0024837363**
Gm23287	0,2530810829	0,001374245
Reg3g	0,2523846645	0,0099252489
Arid1b	0,2148935625	0,0078226139
Fscn1	0,2061316688	0,0087255421
Skor1	0,170660843	0,003380201
**Wnt8b**	**-0,2062715433**	**0,0087604517**
Plxna4os2	-0,2324920865	0,009206205
Npepps	-0,243807535	0,0077846188
Fbxo18	-0,244507896	0,0089366575
Srrm2	-0,2534967529	0,0009093718
Nup205	-0,2554291291	0,0044950192
Zkscan3	-0,2830899347	0,0100248199
Dock7	-0,2946875078	0,000215144
Hbb-y	-0,3001813086	0,0089660215
Gm9531	-0,3061272885	0,0095711757
Optc	-0,3094303304	0,0014856541
Snx32	-0,3219456583	0,0048618493
Kdm6a	-0,3269529612	0,0014868219
1700048O20Rik	-0,3439046943	0,0030767538
Clint1	-0,3485911948	0,0024351027
