# Supplementary figures and images for: Nkx2.9 Contributes to Mid-Hindbrain Patterning by Regulation of mdDA Neuronal Cell-Fate and Repression of a Hindbrain-Specific Cell-Fate
Source: Int J Mol Sci. 2021 Nov 23;22(23):12663. doi: 10.3390/ijms222312663 (PMC8658040; doi:10.3390/ijms222312663)

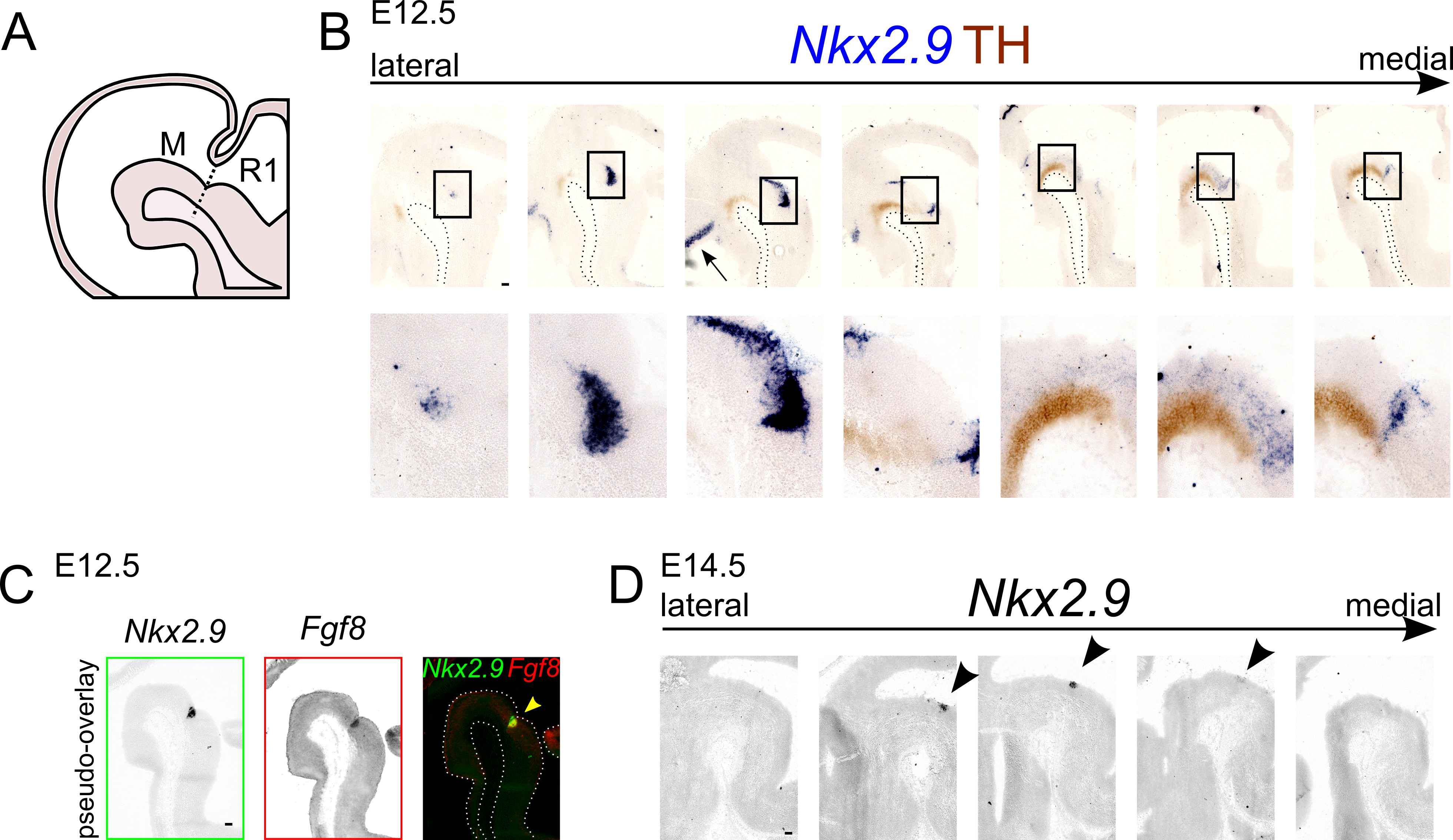

Supplement: Supplementary file 1 [file ijms-22-12663-s001.zip › FigS2.tiff]

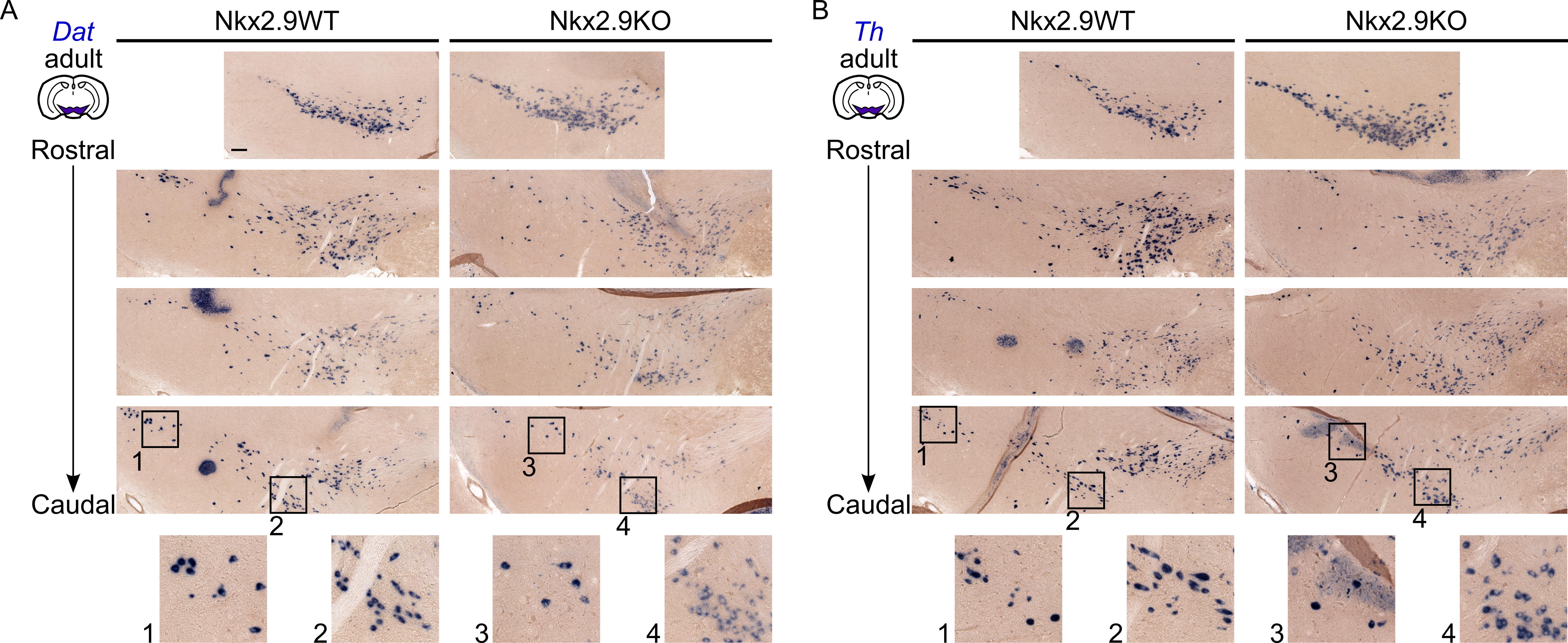

Supplement: Supplementary file 1 [file ijms-22-12663-s001.zip › SupplFigS1.tiff]
